# The role of dislocation-solute interactions on the creep behaviour of binary Mg–RE alloys

**DOI:** 10.1038/s41598-021-82517-5

**Published:** 2021-02-03

**Authors:** Jing Li, Jialin Wu, Li Jin, Mert Celikin, Fenghua Wang, Shuai Dong, Jie Dong

**Affiliations:** 1 National Engineering Research Center of Light Alloy Net Forming and State Key Laboratory of Metal Matrix Composite, School of Materials Science and Engineering, Shanghai Jiao Tong University, Shanghai, 200240 China; 2 I-Form Advanced Manufacturing Research Centre, School of Mechanical and Materials Engineering, University College Dublin, Belfield, Dublin 4, Ireland

**Keywords:** Mechanical properties, Metals and alloys

## Abstract

The effect of dislocation-RE atoms interactions on the creep behaviour has been studied via creep testing and HAADF-STEM analysis of two extruded alloys; Mg–0.5Ce and Mg–2Gd (wt%). Almost no Ce atoms are detected in the Mg matrix due to the low solid solubility and faster diffusion rate in as-extruded condition. However, Gd solute segregations are observed along dislocations and hexagonal dislocation patterns. Such segregations can not only pin the dislocation motion and enhance the creep strengthening via dislocation patterns, but also lead to dynamic precipitation. Thus, combing with the stress exponent values, the transition of creep mechanism between Mg–0.5Ce alloys and Mg–2Gd alloys has been found and dislocation-Gd atoms interactions are determined to be the main factor for superior creep resistance of Mg–2Gd alloys.

## Introduction

Rare earth (RE) elements are intentionally added to pure Mg to modify the mechanical properties^1–4^. If the RE concentration is lower than its solid solubility in pure Mg, the strengthening effect ordinarily comes from the solid solution strengthening, which is conventionally attributed to the elastic interactions between dislocations and local lattice strain caused by RE atoms^5^. However, at high temperatures, the large differences in the atomic radii between RE and Mg atoms can also lead to solute segregation around dislocation cores. Such solute clusters or orderings can exert a drag force on the gliding dislocations, slow down the dislocation motion and thus tailor the macroscopic properties of Mg alloys^6–9^. One typical example regarding such interactions is the Portevin–Le Chatelier (PLC) effect^10^. Within particular temperature and strain rate ranges, mobile dislocations can be continuously pinned and unpinned by RE solute atmospheres and ultimately exhibiting serrations in the flow curve. Zhu et al.^11^ have reported that the PLC effect can not only lead to the negative sensitivity of stress to strain rate but also a plateau in the temperature dependent yield and ultimate strength. Moreover, dislocation substructures can serve as templates for the segregation of RE atoms at high temperatures^12^. Li et al.^13^ discovered the hexagonal patterning Gd nanofibers in extruded Mg–Gd binary alloys and illustrated that these nano structures can potentially modify the mechanical performance by tunning the work hardening behaviour. Therefore, it is of importance to pay attention to the interactions between dislocations and RE atoms as well as their effect on the macroscopic properties, particularly for the plastic deformation at high temperatures.

Limited creep resistance has been regarded as one of the principal hurdles which are restricting the widespread applications of Mg alloys^14,15^. One critical strategy enhancing the creep performance is to alloy Mg with RE elements. To date, solid solution and dynamic precipitation strengthening, if possible, are the two main hardening mechanisms which are responsible for the appealing creep properties^16–18^. Mo et al.^19^ also tried to relate the superior high temperature behavior of binary Mg–RE solid solutions to the short-range order strengthening effect. However, the nature of interactions between dislocations and RE solutes is still ambiguous despite the studies available that have successfully used modelling to shed light into the effects of RE elements^20,21^. There is a lack of experimental data on dislocation-RE solute interactions during creep as it is challenging to isolate the effect of solutes only where grain size and precipitates are important factors as well. In this study, we investigated the dislocation-solute interactions in two binary Mg–0.5Ce and Mg–2Gd (wt%) alloys crept at 200 °C, using high-angle annular dark-field scanning transmission electron microscopy (HAADF-STEM) technique to obtain atomic scale information. Ce and Gd elements are chosen here because they are the most common REs and have different solid solubility limits in pure Mg, i.e., 0.74 wt% and 3.82 wt%, respectively, at 200 °C^20^. The results might provide valuable information for better understanding of strengthening mechanism for solid solution Mg–RE alloys with enhanced creep resistance and shed light for creep resistant Mg alloy design.

## Methods

Mg–0.5Ce and Mg–2Gd alloys ingots were solution treated at 520 °C for 8 h, followed by quenching into water, and then extruded at 350 °C. Dog-bone tensile samples (with a gage length of 15 mm and cross-sectional dimension of 3.5 × 2.2 mm^2^) and flat tensile creep samples (with a gage length of 25 mm and cross-sectional dimension of 6 × 2 mm^2^), in accordance with the ASTM-E21 and ASTM-E139 standards, respectively, were machined out of the two as-extruded bars along the extrusion direction (ED). High temperature tensile tests were performed at 150–250 °C with a strain rate of 0.001 s^−1^. Tensile creep tests were carried out at temperature of 200 °C and applied stress of 30–50 MPa for Mg–0.5Ce alloys and 50–90 MPa for Mg–2Gd alloys, respectively. Prior to SEM, EDS and EBSD examinations, the sample surfaces were prepared by SiC paper, 6 μm, 3 μm, 1 μm diamond suspension and OPS suspension. The step size used in EBSD tests was 0.5 μm. Data obtained from the EBSD tests were analyzed using TSL OIM software. TEM observation was carried out using JEM 2100F TEM at 200 kV and HAADF-STEM mode of JEOL-ARM200F microscope with a probe-forming lens corrector. All TEM samples were water quenched immediately after creep tests and then prepared by dimple grinding and ion-milling using a Gatan precision ion polishing system (PIPS II MODEL 695).

## Results and discussion

**Figure 1:**
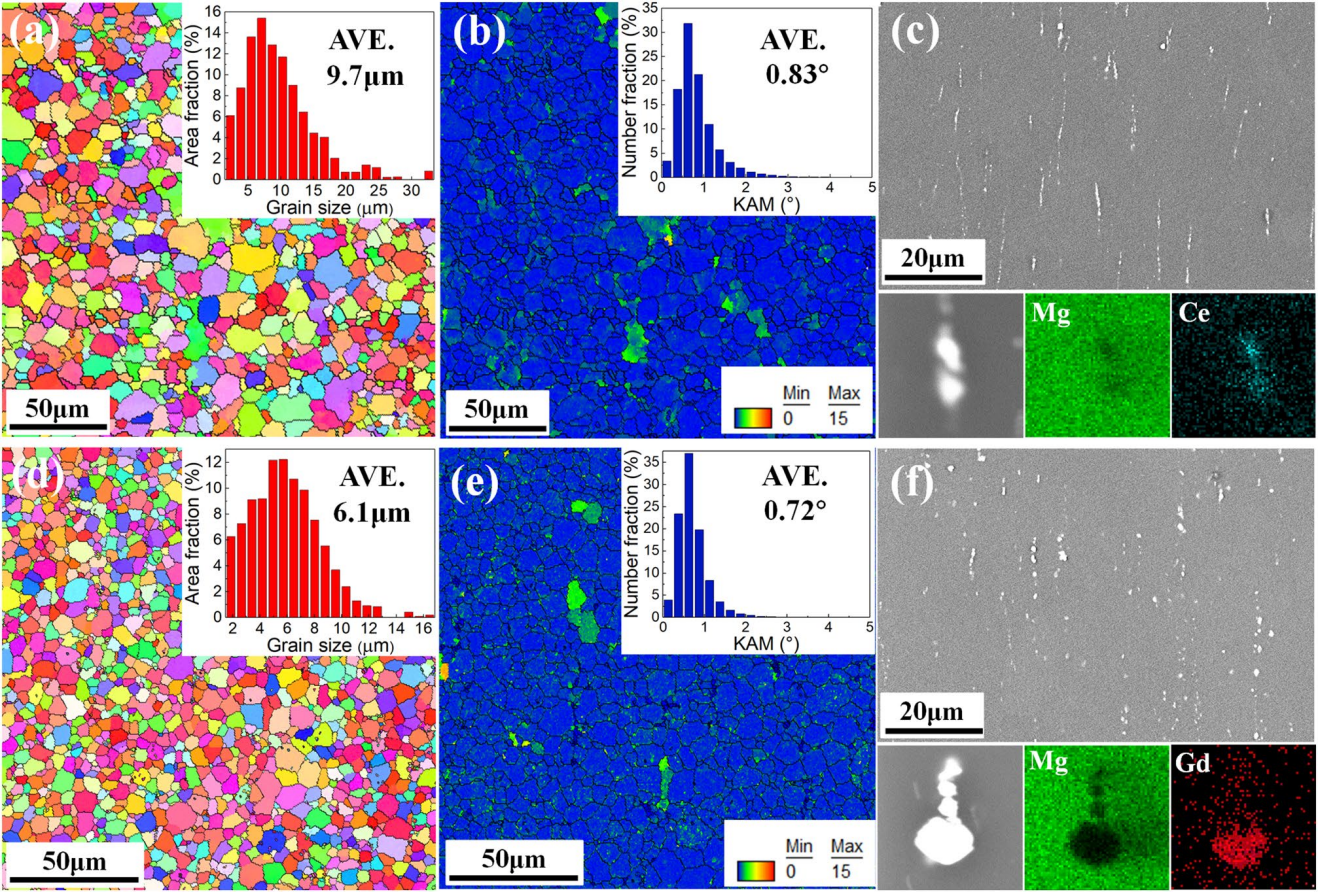
Microstructure of extruded Mg–0.5Ce (the first row) and Mg–2Gd alloys (the second row) before creep tests: (**a**,**d**) EBSD orientation maps, (**b**,**e**) KAM images, (**c**,**f**) SEM images and EDS analysis.

Figure 1 shows the initial microstructure of the as-extruded Mg–0.5Ce and Mg–2Gd alloys. As can be seen, the two alloys exhibit similar average grain size, i.e., 9.7 μm for Mg–0.5Ce and 6.1 μm for Mg–2Gd alloys, respectively. (Fig. 1a,d). The kernel average misorientation (KAM) plot in Fig. 1b,e show that both Mg–0.5Ce and Mg–2Gd alloys have low residual strain. KAM value has a relationship with geometry necessary dislocations (GNDs) and hence can refer to the residual strain^21^. Figure 1c,f show the SEM and EDS results for Mg–0.5Ce and Mg–2Gd alloys. During extrusion at high temperature, RE-based dynamic precipitation occurred in both alloys since those secondary precipitations are not found in the alloys after solution treatment (Fig. S1) and are parallel to the ED. Dynamic precipitates formed are most probably Mg_12_Ce (Mg–0.5Ce) and Mg_5_Gd (Mg–2Gd) as they are the most stable secondary phases observed in previous studies^22,23^.

**Figure 2:**
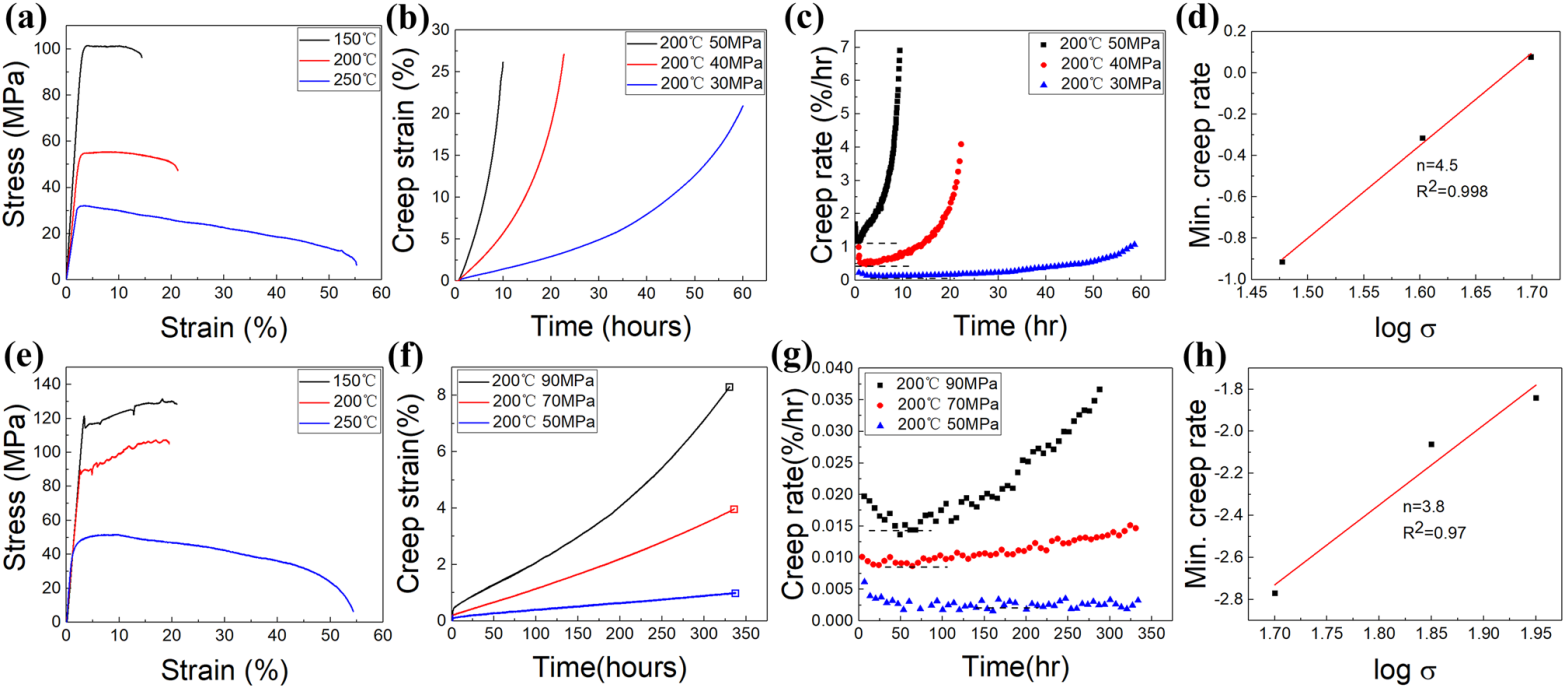
Mechanical behaviours of Mg–0.5Ce (the first row) and Mg–2Gd alloys (the second row) at high temperature: (**a**,**e**) tension curves at the temperature ranging from 150 to 250 °C, (**b**,**f**) tension creep curves at 200 °C and various applied stress, in which squares mark the point that testing is terminated artificially, (**c**,**g**) creep rate vs. time images, (**d**,**h**) log steady state strain rate plotted as a function of log stress.

Figure 2a,e show the tensile curves of the two alloys at the temperatures ranging from 150 to 250 °C. Smooth flow curves are observed for the Mg–0.5Ce alloys. However, the Mg–2Gd alloys exhibit serrated flow at the temperatures of 150 °C and 200 °C and the serrations disappear at 250 °C. Extensive studies have illustrated that those serrated curves at high temperature are known as PLC effect, caused by the drag force of diffusion solute atoms which pin the dislocations temporarily and then raise the stress to reactive them^10,11^. For the purpose of investigating the interactions between dislocations and solute atoms during creep process, the temperature of 200 °C is chosen to carry out the creep tests since at this temperature serrated flow curve is detected for the Mg–2Gd alloys but not for the Mg–0.5Ce alloys. Additionally, the yield stress for the two alloys at 200 °C is different, i.e., 55 MPa for Mg–0.5Ce alloys and 92 MPa for Mg–2Gd alloys. For comparison, the applied stress which has similar ratio to the yield stress for the two alloys at 200 °C is selected for creep tests, which is listed in Table.1.

**Table 1:** Summary of the creep properties of Mg–0.5Ce and Mg–2Gd alloys.

Samples	Temperature ( °C)	Applied stress (MPa)	Ratio of the applied stress to the yield stress at 200 °C	Minimum creep rate (%/h)	Creep life (h)
Mg–0.5Ce alloys	200	30	0.55	0.14	60
		40	0.73	0.51	23
		50	0.91	1.20	10
Mg–2Gd alloys	200	50	0.54	0.0025	> 330
		70	0.76	0.0091	> 330
		90	0.98	0.0144	> 330

The creep responses of the two investigated alloys are displayed in Fig. 2b,f by strain vs. time plots. A remarkable improvement in creep resistance is found for the Mg–2Gd alloys in comparison to the Mg–0.5Ce alloys. For example, at the temperature of 200 °C and the ratio of applied stress to yield stress of ~ 0.55, the Mg–0.5Ce alloys failed at 60 h with a strain of ~ 21% but the Mg–2Gd alloys remain intact after lasting 350 h showing a strain of ~ 1%. It can also be seen from Fig. 2c,g that the minimum creep rates of Mg–2Gd alloys are almost two orders of magnitude lower than that of Mg–0.5Ce alloys. Figure 2d,h show the log minimum creep rate vs. log stress for Mg–0.5Ce and Mg–2Gd alloys. As can be seen, the stress exponent decreases from 4.5 for the Mg–0.5Ce alloys to 3.8 for the Mg–2Gd alloys. It has been widely accepted that dislocation gliding and climbing is most likely the dominant mechanism when the stress exponent is in the range of 4–7 but solute drag dislocation gliding takes the controlling role as the stress exponent reduces to 3^24–26^. The values of all critical creep properties are summarized in Table.1.

**Figure 3:**
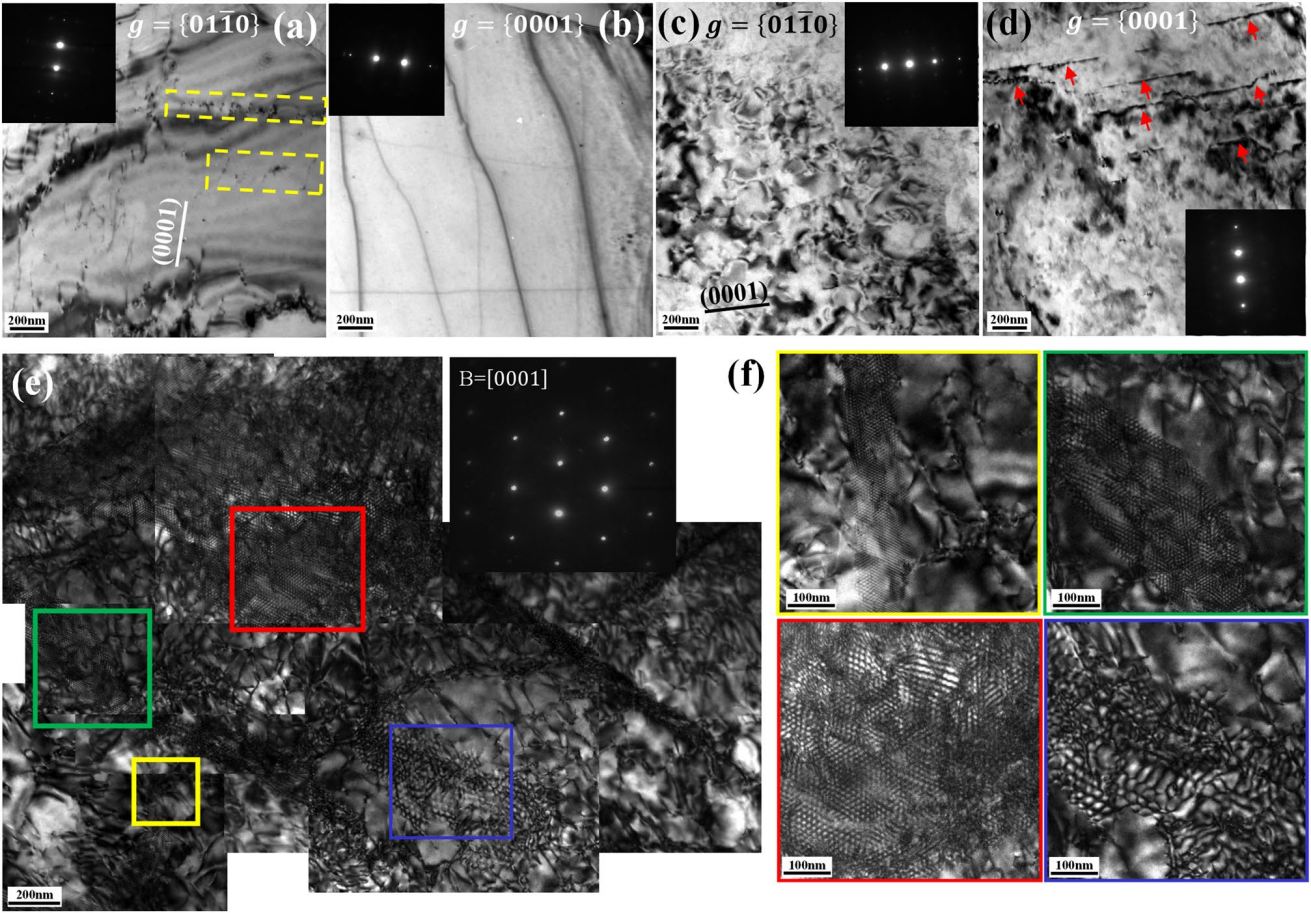
TEM images taken near the minimum creep rates, i.e., after ~ 3 h at the applied stress of 40 MPa for Mg–0.5Ce alloys and ~ 60 h at 70 MPa for Mg–2Gd alloys: (**a**,**b**) two beam conditions for the Mg–0.5Ce alloys, where dislocation climbing is marked by the yellow squares, (**c**,**d**) two beam conditions for Mg–2Gd alloys, in which the <c> dislocations are marked by the red arrows, (**e**) the hexagonal dislocation patterns observed along the [0001] axis in the Mg–2Gd alloys, (**f**) corresponding enlarged images marked by the squares in (**e**).

To further probe the creep mechanisms, in-depth dislocation analysis is conducted in the Mg–0.5Ce and Mg–2Gd alloys after terminating the creep tests near their minimum creep rates, i.e., after ~ 3 h at the applied stress of 40 MPa for Mg–0.5Ce alloys and after ~ 60 h at 70 MPa for Mg–2Gd alloys, respectively. Figure 3a,b show the TEM images taken under the two beam conditions for the Mg–0.5Ce alloys. The dislocations are identified as < a > type dislocations based on the g·b analysis that they are visible under $g=\left\{01\stackrel{-}{1}0\right\}$ and invisible under $g=\left\{0002\right\}$. Moreover, most of the dislocation lines lying along the basal plane trace are basal <a> dislocations and several dislocation segments extending out of the basal plane suggest that cross slip occurs. Additionally, a few short <a> dislocations piling up in arrays and parallel to each other, as marked by the yellow squares, are detected in the Mg–0.5Ce alloys, which are probably resulted from the dislocation climbing. Thus, dislocation gliding and climbing are the main creep mechanisms of Mg–0.5Ce alloys, agreeing well with the determined stress exponent.

TEM images taken from the Mg–2Gd alloys are shown in Fig. 3c,d. In addition to basal <a> dislocations, some <c> type dislocations, as marked by the red arrows, are also recognized in the Mg–2Gd alloys as they are visible under $g=\left\{0002\right\}$ and invisible under $g=\left\{01\stackrel{-}{1}0\right\}$. <c> dislocations can come from two sources, either the activated <c> slips or the dissociation of <c+a> dislocations^27,28^. When <c+a> dislocations dissociate into <a> and <c> dislocations, basal <a> dislocations can glide easily to annihilate with the opposite signed <a> dislocations and hence <c> dislocations are left^29^. In addition, from Fig. 3c, higher percentage of cross slip is observed in the Mg–2Gd alloys in comparison to the Mg–0.5Ce alloys as most of the <a> dislocations found in the Mg–2Gd alloys are lying out of the basal plane. This may be partly due to the higher applied stress, but the solute drag force may also play a role. Since the drag force exerted by the solid atmosphere affects the mobility of basal dislocations, dislocations may cross slip to non-basal planes under the thermal activation to relieve the stress concentration. However, the interactions between dislocations and RE atoms need to be further confirmed at atom scale, which will be addressed later in this work.

Moreover, viewed from the [0001] axis as shown in Fig. 3e, hexagonal dislocation patterns, which extend a few microns in size, are detected in the secondary creep stage of Mg–2Gd alloys. From the corresponding enlarged images in Fig. 3f, we can see that such dislocation patterns exhibit different inter-spacings ranging from 4 to 40 nm. Hexagonal dislocation patterns have also been found in pure Mg after compression at 400 °C and are related to the self-assemble of a set of parallel <c> -screw dislocation dipoles^29^. <c> type dislocations are observed in the Mg–2Gd alloy by using two beam conditions, as shown in Fig. 3d. No such dislocation patterns found in the Mg–0.5Ce alloys are also understandable since there are few <c> dislocations observed. Moreover, we should note that such dislocation patterns are not observed in the first and third creep stages of Mg–2Gd alloys as well. In this sense, a strong forest strengthening exerted by such dislocation patterns would be expected. This can be related to the unique orientation of those hexagonal patterned <c> dislocations, which is lying along the [0001] axis, and hence have a high possibility of inhibiting the easy-gliding basal slip. In addition, the segregation of Gd clusters which can form the Gd-nano fibers would also possibly play a role in, as the Gd segregations were previously reported in the extruded Mg–1Gd alloys^13^.

**Figure 4:**
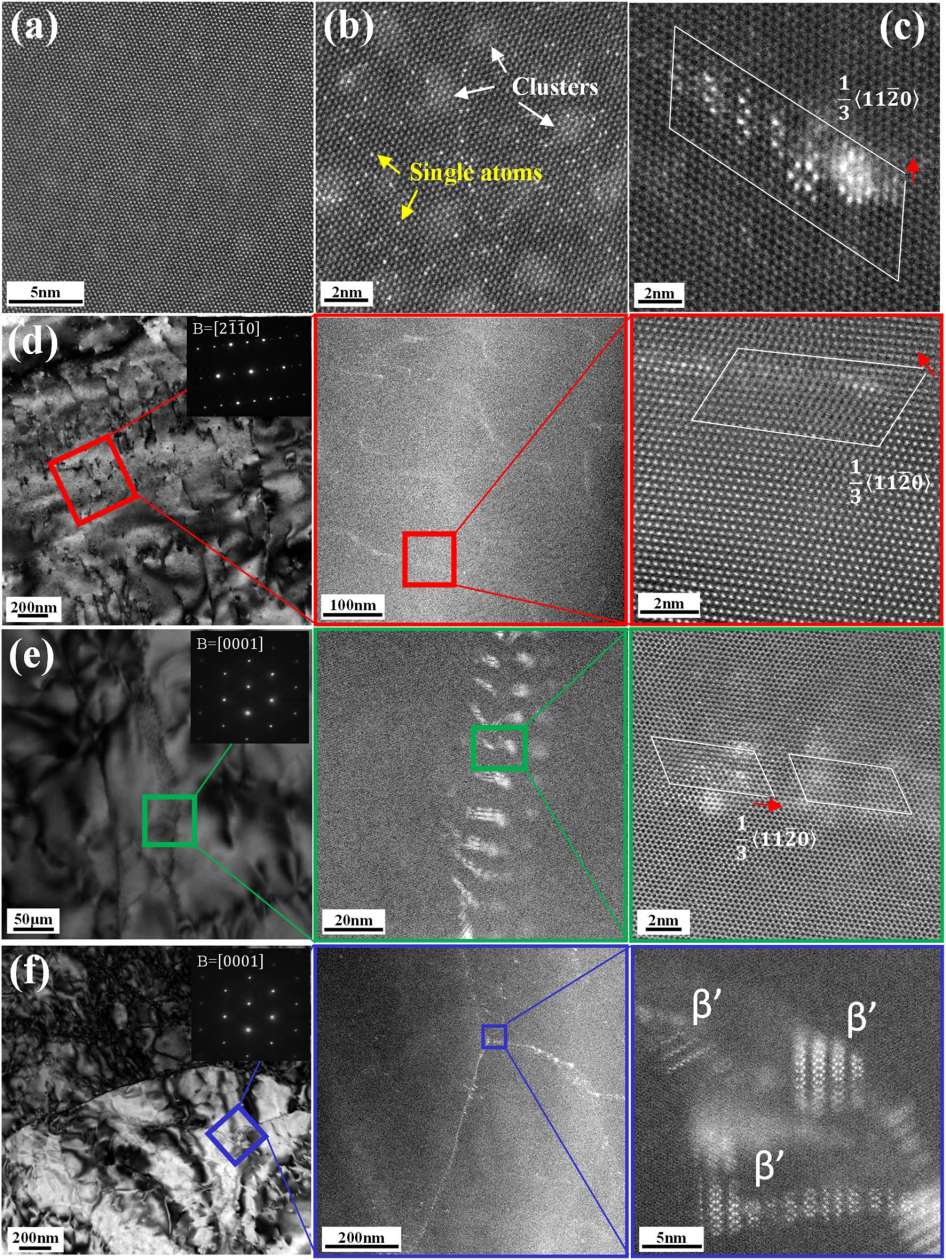
HAADF-STEM images taken near the minimum creep rates, i.e., after ~ 3 h at the applied stress of 40 MPa for Mg–0.5Ce alloys and ~ 60 h at 70 MPa for Mg–2Gd alloys: (**a**) Mg–0.5Ce alloys recorded along the $[2\stackrel{-}{1}\stackrel{-}{1}0]$ zone axis, (**b**–**f**) Mg–2Gd alloys.

Due to the large difference between the atomic numbers of the constituent elements (Mg: 12, Ce: 58, Gd: 64), it is easy to detect the heavy Ce and Gd atoms in the Mg matrix by using HAADF-STEM^30,31^. The investigated samples taken from the Mg–0.5Ce and Mg–2Gd alloys are under the same creep conditions as those of TEM samples, respectively. Figure 4a shows the atomic-resolution HAADF-STEM image of the Mg–0.5Ce alloys recorded along the $[2\stackrel{-}{1}\stackrel{-}{1}0]$ zone axis. Surprisingly, no bright Ce atoms are detected in the Mg matrix. Another grain has also been investigated along the [0001] axis, still no Ce atoms are observed (Fig. S2). However, Gd atoms can be seen in the Mg matrix of the Mg–2Gd alloys, either as single atoms or as clusters (Fig. 4b). The lack of Ce atoms is probably due to the lower Ce content (0.5 wt%) and faster diffusion rates of Ce solute than Gd^32^. Faster diffusion rate allows the formation and growth of Ce-based dynamic precipitates during extrusion at 350 °C leading to the depletion of Ce atoms in the Mg matrix prior to the creep testing. Hence, it can be stated that Mg–0.5Ce alloys would show similar creep behaviour with pure Mg when the deformation is controlled via dislocation-based creep processes. Crossland and Jones^33^ conducted creep tests on pure Mg and found that the stress exponent n was 4.4 at the temperature of 200 °C and in the stress range of 17–31 MPa. Given that the yield stress of pure Mg at 200 °C is 25–32 MPa^34,35^, represents similar ratios between stresses used during creep testing and the high temperature yield strength with the ones used in present study for Mg–0.5Ce and Mg–2Gd alloys. Vagarali and Langdon^36^ also reported a stress exponent of 5.5 under similar creep testing conditions. Thus, dislocation gliding and climbing are also suggested as the main creep mechanisms for pure Mg, similar with that of Mg–0.5Ce alloys.

Enlarged image in Fig. 4c shows a dislocation with the Burger vector of $\frac{1}{3}\langle 11\stackrel{-}{2}0\rangle$ in the Mg–2Gd alloys. Bright contrast at the dislocation core indicates the G.P. zone consisting of Gd atoms, which are arranged as hexagonal and zig-zag chains. Such interactions between dislocations and Gd atoms can also be observed along the $[2\stackrel{-}{1}\stackrel{-}{1}0]$ axis. As shown in Fig. 4d, a continuous bright contrast is seen along the dislocations. Detailed Burger circuit analysis further identifies the dislocations as <a> type. As illustrated extensively, the strain field associated with dislocations should be the reason for the segregation of Gd atoms^8,37,38^. Under the consideration of reducing the elastic strain of individual solute atoms, the observation of G.P. zones rather than Gd clusters can also be understood^39^. Figure 4e shows the segregation of Gd atoms in the hexagonal dislocation patterns, as confirmed by the bright intensity in the corresponding HAADF-STEM images. Burger’s loop analysis was used to recognize the dislocation type, which shows such dislocation patterns not only consist of <a> dislocations but also the dislocations with closed Burgers circuits. Combining experimental results and model calculation, Liu et al.^29^ illustrated those dislocations showing no edge components are <c> -screw dislocations. Li et al.^13^ further observed such Gd segregation along the $[11\stackrel{-}{2}0]$ axis and found that Gd segregation templated to the dislocation patterns can finally lead to the formation of the Gd nano-fibers with the <c> rod shape. Based on above experimental results, the interactions between dislocations and Gd atoms are confirmed in the Mg–2Gd alloys, either in the way of segregating along the dislocations or into the hexagonal dislocation patterns. The stress exponent is valid for the Mg–2Gd alloys and solute drag gliding is verified to be the main creep mechanism.

Therefore, it is found that the creep mechanism changes from dislocation gliding and climbing for the Mg–0.5Ce alloys to solute drag dislocation gliding for the Mg–2Gd alloys even under the same creep temperature and the ratio of the applied stress to yield stress for the two alloys are similar. In this case, apart from the solid solute strengthening offered by higher concentration of Gd atoms, dislocation-Gd atoms interactions also play a critical role in the superior creep resistance of the Mg–2Gd alloys. Firstly, Gd segregation would exert a strong pinning effect on the dislocation motion and reduce the creep rate dramatically. Secondly, the strengthening effect achieved from the hexagonal dislocation patterns can be further enhanced by Gd segregation. On one side, such segregation can reduce the dislocation core energy and then improve the dislocation patterns’ stability^40^. On the other side, the formed Gd nano fibers can act as effective obstacles for the easy-gliding basal dislocations since they are parallel to the [0001] axis^13^. Moreover, such interactions would lead to dynamic precipitation. As seen in Fig. 4f, ${\beta }^{^{\prime}}$ phases are formed along the low angle grain boundary. In this case, dynamic precipitates also offer a certain strengthening effect for the higher creep resistance shown in the Mg–2Gd alloys.

In summary, after comparing the creep behavior of extruded Mg–0.5Ce and Mg–2Gd alloys under the temperature of 200 °C and the similar ratio of the applied stress to the yield stress, a transition of the creep mechanism from dislocation climb for the Mg–0.5Ce alloys to solute drag dislocation glide for the Mg–2Gd alloys has been verified. Low solid solubility and faster diffusion rate of Ce lead to almost no Ce atoms left in the Mg matrix upon high temperature extrusion. However, the interactions between dislocations and Gd atoms are detected in the as-extruded Mg–2Gd alloys. Such interactions can not only exert a drag effect on the dislocation motion and enhance the strengthening effect offered by the hexagonal dislocation patterns, but also lead to the formation of dynamic precipitation. In this case, dislocation-Gd atoms interactions are crucial for the high creep resistance of the Mg–2Gd alloys.

## Supplementary Information

Supplementary Information
