# Supplementary material for: The role of dislocation-solute interactions on the creep behaviour of binary Mg–RE alloys
Source: Sci Rep. 2021 Feb 3;11:2860. doi: 10.1038/s41598-021-82517-5 (PMC7859206; doi:10.1038/s41598-021-82517-5)
Supplement: Supplementary file 1 — Supplementary Information [file 41598_2021_82517_MOESM1_ESM.docx]

**Supplementary Material**

**The Role of Dislocation-solute Interactions on the Creep Behaviour of**

**Binary Mg-RE Alloys**

Jing Li^a^, Jialin Wu^a^, Li Jin^a,^ *, Mert Celikin^b^, Fenghua Wang^a^, Shuai Dong^a^, Jie Dong^a^

*^a^ National Engineering Research Center of Light Alloy Net Forming and State Key Laboratory of Metal Matrix Composite, School of Materials Science and Engineering, Shanghai Jiao Tong University, Shanghai 200240, China.*

*^b^ I-Form Advanced Manufacturing Research Centre, School of Mechanical and Materials Engineering, University College Dublin, Belfield, Dublin. 4, Ireland.*

1. Fig.S1-Fig.S2

* Corresponding author:

**Li Jin**, E-mail address: [j_jinli@sjtu.edu.cn](mailto:j_jinli@sjtu.edu.cn), Tel: 86-21-34203052, Fax: 86-21-34202794

**1. Fig.S1-Fig.S2**

**
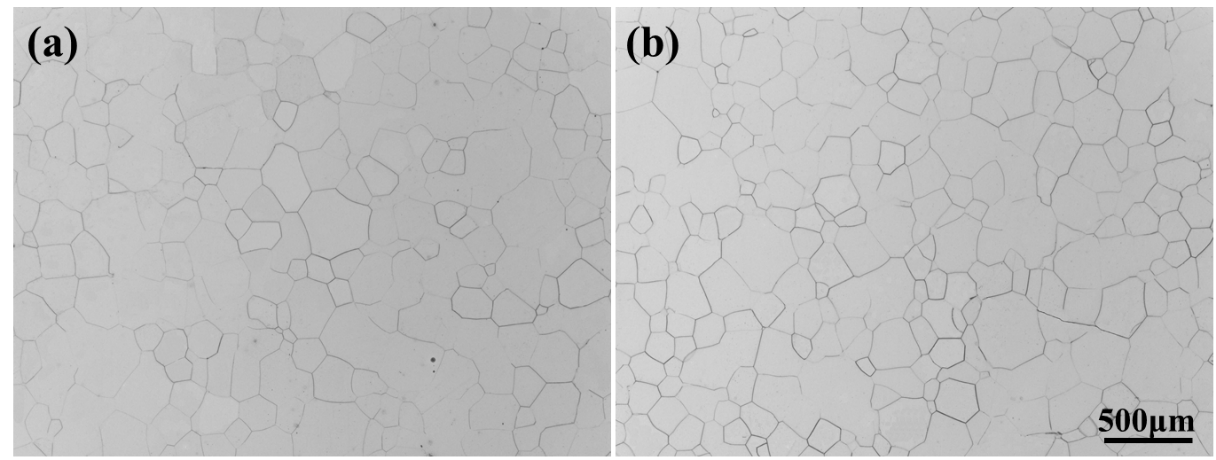
**

**Fig. S1** Optical images of Mg-0.5Ce and Mg-2Gd alloys after solid solution treatment.


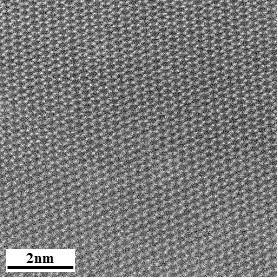


**Fig. S2** HAADF-STEM image taken near the minimum creep rates of Mg-0.5Ce alloys along the [$0001$] zone axis.
